# Denervated muscle fibers induce mitochondrial peroxide generation in neighboring innervated fibers: Role in muscle aging

**DOI:** 10.1016/j.freeradbiomed.2017.07.017

**Published:** 2017-11

**Authors:** Natalie Pollock, Caroline A. Staunton, Aphrodite Vasilaki, Anne McArdle, Malcolm J. Jackson

**Affiliations:** MRC – Arthritis Research UK Centre for Integrated Research into Musculoskeletal Ageing (CIMA), Department of Musculoskeletal Biology, Institute of Ageing and Chronic Disease, University of Liverpool, Liverpool L7 8XL, UK

**Keywords:** Hydrogen peroxide, Denervation, Skeletal muscle, Sarcopenia

## Abstract

Disruption of neuromuscular junctions and denervation of some muscle fibers occurs in ageing skeletal muscle and contribute to loss of muscle mass and function. Aging is associated with mitochondrial dysfunction and loss of redox homeostasis potentially occurs through increased mitochondrial generation of reactive oxygen species (ROS). No specific link between increased mitochondrial ROS generation and denervation has been defined in muscle ageing. To address this, we have examined the effect of experimental denervation of all fibers, or only a proportion of the fibers, in the mouse tibialis anterior (TA) muscle on muscle mitochondrial peroxide generation. Transection of the peroneal nerve of mice caused loss of pre-synaptic axons within 1–3 days with no significant morphological changes in post-synaptic structures up to 10 days post-surgery when decreased TA mass and fiber size were apparent. Mitochondria in the denervated muscle showed increased peroxide generation by 3 days post-transection. Use of electron transport chain (ETC) substrates and inhibitors of specific pathways indicated that the ETC was unlikely to contribute to increased ROS generation, but monoamine oxidase B, NADPH oxidase and phospholipase enzymes were implicated. Transection of one of the 3 branches of the peroneal nerve caused denervation of some TA muscle fibers while others retained innervation, but increased mitochondrial peroxide generation occurred in both denervated and innervated fibers. Thus the presence of recently denervated fibers leads to increased ROS generation by mitochondria in neighboring innervated fibers providing a novel explanation for the increased mitochondrial oxidative stress and damage seen with aging in skeletal muscles.

## Highlights

• Denervation causes increased mitochondrial peroxide release in muscle fibers.
• Increased mitochondrial peroxide release precedes loss of skeletal muscle mass.
• Increased peroxide release is not due to increased superoxide generation by ETC complexes.
• Increased peroxide release occurs in both denervated and neighboring innervated fibers.
• Data provide an explanation for increased mitochondrial peroxide seen in aging muscle.

## Graphical abstract

**Figure:**
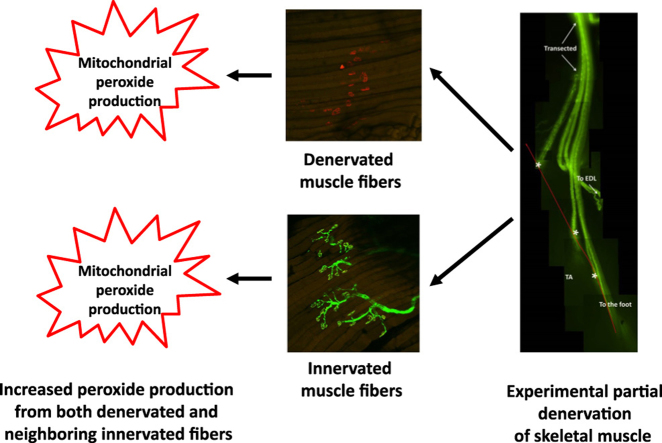

## 1 Introduction

During aging a decrease in muscle mass due to loss and atrophy of muscle fibers together with reduced muscle strength is apparent [1], [2] and is a major contributor to frailty in the elderly [3]. These changes in muscle are accompanied by degeneration in motor neurons and neuromuscular junctions (NMJ). With increasing age there is a decline in motor neuron number and morphological breakdown of some NMJ in humans [4] and rodents [5]. Age-related structural changes within the NMJ include partial or complete loss of pre-synaptic input, blebbing of the peripheral axons and fragmentation of post-synaptic acetylcholine receptor (AChR) clusters [6], [7], [8]. Such changes may primarily occur in the peripheral axons since minimal loss of motor neuron cell bodies in the lumbar spinal cord has been reported in aged rodents [9]. Throughout life, cycles of denervation and re-innervation of individual muscle fibers occur [10] and with advancing age these processes slow and may eventually breakdown [11], [12] with an accumulation of denervated fibers [8].

Reactive oxygen species (ROS) have key roles in cell signalling linked to cell differentiation and survival [13], [14] and in skeletal muscle the production of ROS during contractile activity mediates adaptive responses to contractions through redox-signalling processes that activate key transcription factors [15], [16]. Increased generation of ROS by muscle mitochondria has been implicated in age-related oxidative stress and loss of muscle fibers [17]. Recent studies have also linked oxidative stress to loss of muscle mass and function since mice lacking SOD1 (Cu,Zn superoxide dismutase) [18] show accelerated, age-related neuromuscular degeneration [2].

The mechanisms underlying the age-related increase in ROS generation by muscle mitochondria are unknown although increased generation of superoxide by electron transport chain (ETC) complexes has been implicated. Experimental denervation of skeletal muscle has also been shown to cause large increases in the generation of hydrogen peroxide (H_2_O_2_) and other peroxides by mitochondria from denervated rodent skeletal muscle although the potential role of this in aging has not been explored [19]. During aging only a minor proportion of muscle fibers lack functional innervation, but we hypothesised that the lack of innervation of some fibers would lead to an increase in mitochondrial peroxide generation that would be sufficient to influence the integrity of neighboring innervated fibers. The current study has used a model of partial denervation of the tibialis anterior (TA) muscle to demonstrate that the presence of recently denervated fibers in a skeletal muscle induces increased peroxide generation by mitochondria in the denervated fibers and in neighboring fully innervated fibers by a mechanism that does not require increased superoxide generation by ETC complexes and these data provide a novel explanation for the increased mitochondrial oxidative stress and damage seen in aging skeletal muscle.

## 2 Materials and methods

### 2.1 Animals

Male B6.Cg-Tg (Thy1-YFP) mice aged 4–8 months (adult) or 24 months (old) were bred and maintained under SPF conditions. These animals express yellow fluorescent protein (YFP) only in neuronal cells with no toxic effects [6]. The mice were fed ad libitum on standard laboratory diet and subjected to a 12 h light, 12 h dark cycle. Procedures were performed in accordance with UK Home Office Guidelines under the UK Animals (Scientific Procedures) Act 1986 and received ethical approval from the University of Liverpool Animal Welfare Committee. Animals were anaesthetised by inhalation of isoflurane, the hind limbs were shaved and animals were administered 100 µl buprenorphine (0.3 mg/ml) prior to surgery. Surgical procedures were carried out under aseptic conditions with animals maintained under gas anaesthesia for the duration of the procedure. A cyan (ex490-515 nm, ex 550LP) fluorescence adaptor (Nightsea, Lexington, USA) for stereo-dissection microscope was utilised throughout the procedure to allow visualisation of the detailed nerve structure.

### 2.2. Surgical procedures

#### 2.2.1 Full denervation of the TA muscle

In order to fully denervate the TA muscle, the peroneal nerve was fully transected as it runs over the lateral head of the gastrocnemius and a small section was removed. A sham procedure, involving exposure of the nerve without transection, was carried out on the contralateral limb. The exterior wound was sutured and animals were allowed to recover for 1, 3, 7 or 10 days.

#### 2.2.2. Partial denervation of the TA muscle

Prior to entering the flexor digitorum longus (FDL), the common peroneal nerve divides and branches to innervate the lateral or anterior muscles, the TA and EDL. Multiple terminal nerve branches innervate the TA and can be visualised by separating the FDL from the neighboring posterior muscles and tracing the nerves in an inferior direction. At this point the uppermost branch, which enters the superior portion of the AT, was transected and a small section of the nerve removed (shown in the ex-vivo preparation in Fig. 3a). A sham procedure was performed on the contralateral limb. Animals were allowed to recover for 7 days following surgery.

At the specified time points post nerve transection, animals were killed by a schedule 1 procedure and muscles rapidly excised. The TA was divided for measurement of mitochondrial H_2_O_2_ release in permeablised muscle fibers and imaging (n = 4), or removed intact with the extensor digitorum longus ensuring that the nerve remained attached in order that the innervation could be imaged ex vivo (n = 4).

### 2.3 Assessment of morphological changes in skeletal muscle following denervation

The superior portion of the TA was embedded in OCT (Thermo-Scientific, Cheshire, UK) and frozen in liquid nitrogen cooled isopentane. 12 µm transverse sections were obtained on a cryostat (Leica CM1850, Germany) for analysis of fiber CSA and NCAM expression [20], [21]. Samples were fixed onto slides in ice-cold methanol for 15 min before incubation in blocking solution (PBS with 1% triton-X, 5% BSA and 1% goat/donkey serum) for 1 h. Following incubation with rabbit anti-NCAM (Millipore, Billerica, USA) overnight at 4 °C, samples were washed with PBS and incubated with Alexa- 532 donkey anti-rabbit (Invitrogen, Carlsbad, USA). Sections were counter-stained with WGA-488 (5 µM) (Vector Labs, UK) for 15 min at room temperature before vectashield hardset mountant with DAPI (Vector Labs, UK) was added to the slides.

Images covering the whole muscle section were collected using ×20 objective and confocal microscopy (Nikon, Kingston, UK). The images were overlapped to create a montage of the muscle section and image J software (NIH) was used to measure CSA of all muscle fibers.

### 2.4 Mitochondrial H_2_O_2_ release

Small bundles of muscle fibers from the inferior portion of the TA were used to assess the rate of mitochondrial generation of H_2_O_2_ and potentially other peroxides using an amplex red assay. Individual bundles were placed into saponin (200 μM) in relax solution to permeabilise the muscle fibers. The saponin was washed from the tissue through 3 cycles of fresh relax solution with gentle agitation prior to placing the bundles into amplex red solution in black 96 well plates (Corning, Wiesbaden, Germany). Bundles were either incubated with amplex red solution without added substrates to assess state 1 production, or in the presence of ETC substrates or an inhibitor: succinate (5 mM), glutamate-malate (5 mM), rotenone (4 μM) plus succinate (5 mM). Bundles were also treated with inhibitors of other pathways for generation of ROS. We examined the effect of inhibitors of monoamine oxidase B (parglyine, 100 μM) or monoamine oxidase A (chlorgyline, 100 μM), a substrate for monoamine oxidases (tyramine, 30 μM) [22], a NADPH oxidase inhibitor (apocynin, 0.5 mM) [23], a phospholipase A2 inhibitor (arachidonyl trifluoromethyl ketone; AACOCF3, 20 μM) [24] or a xanthine oxides inhibitor (allopurinol, 300 μM) [25]. Fluorescence was followed at 590 nm using a fluorimeter (Fluorstar, BMG Labteck, Germany) at 37 °C. Horseradish peroxidase (HRP, 1 U/ml) was added to the amplex red reagent (80 µM) (Molecular Probes, Eugene, USA), made up in ROS buffer (125 mM KCL, 10 mM Hepes, 5 mM MgCl_2_, 2 mM K_2_HPO_4_) and formation of the fluorescent product resorufin red monitored [26]. SOD (37 U/ml) was added to the mixture to convert any superoxide present into H_2_O_2_ as previously described [27], [28].

In order to assess whether there were regional differences in the rate of mitochondrial H_2_O_2_ release in muscles which had undergone the partial denervation procedure, the inferior portion of the TA was cut into 4 regions as described in the text. Small bundles of fibers (15–25 fibers) from each of the regions were obtained using a scalpel (10 bundles). Once divided the bundles were treated as described above.

### 2.5. Morphological assessment of peripheral axons and AChR

For longitudinal assessment of the integrity of innervation and NMJ morphology, the TA was dissected with the peroneal nerve attached, pinned onto Sylgard plates (Farnell, Preston, UK) and fixed in 10% neutral buffered formalin. The tissues were washed in PBS/1% Triton-X prior to incubation with Alexa-532 conjugated bungarotoxin (Invitrogen) for 30 min. Following 3 × 5 min washing in PBS-tween the muscles were imaged using in-situ fluorescent confocal microscopy (Nikon A1, Kingston, UK).

### 2.6. Statistical analyses

Data are presented as mean±SEM unless stated otherwise. Comparisons were performed by 1-way ANOVA and Tukey or Fisher comparison using Minitab software with P < 0.05 deemed statistically significant.

## 3. Results

### 3.1. Time course of changes following full transection of the peroneal nerve

**Fig. 1:**
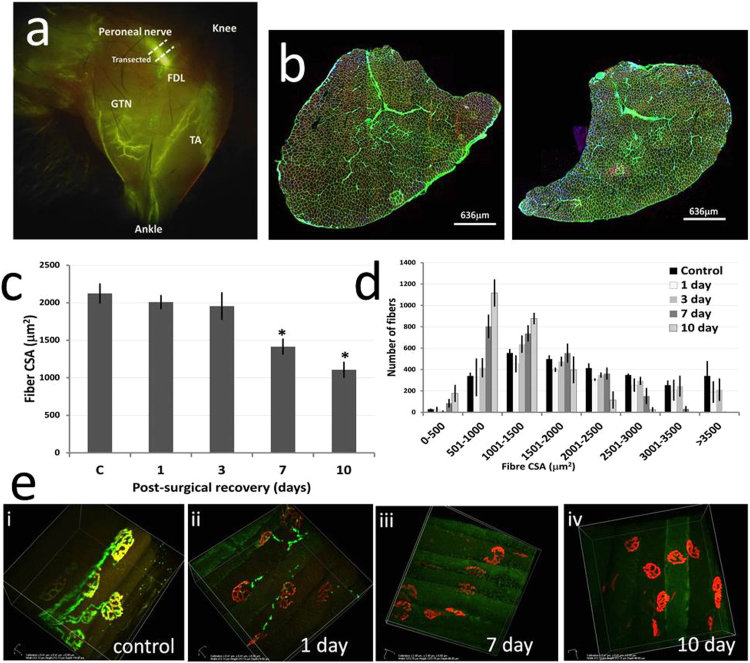
**Effect of peroneal nerve transection on structure of the TA muscle.** Fluorescent image of the lower limb of a Thy1-YFP mouse showing the anatomical structure and the site of peroneal nerve transection (A). Cross-sectional images of a control (left) and denervated (right) TA muscle at 7 days post transection, visualised with WGA (green) and Dapi (blue) (B). The temporal effect of denervation on the mean CSA of fibers in the TA muscle, *P < 0.05 compared with control non-denervated muscle (C). The distribution of fiber cross-sectional areas in the TA muscle (D). Break-down of the NMJs of the TA muscle with time following transection of the peroneal nerve (E). Control muscles show the motor nerves (green) and the motor end plates stained with α-bungarotoxin (red) with pre- and post- synaptic regions perfectly overlapped (Fig. 1E-a). Images following denervation show lack of pre-synaptic input by 1 day post-denervation (1E-b) and by 7 days there is no evidence of pre-synaptic input at the NMJs although the AChRs appear intact (1E-c). By 10 days there is additionally punctate α-bungarotoxin staining in areas distant from the NMJ (1E-d).

Full denervation of the TA muscle was achieved by surgically removing a small section of the peroneal nerve prior to entry into the muscle (Fig. 1a). The mean fiber cross sectional area (CSA) in the denervated TA was decreased in comparison with that from control TA muscle with the decrease significant at 7 days post-denervation (Fig. 1b, c). Analysis of the distribution of fiber areas indicted that denervation induced a decrease in the number of larger fibers (greater than 3000 µm^2^) at 7 and 10 days post-transection with an increase in the number of smaller fibers (500–1500 µm^2^) compared with control muscle (Fig. 1d).

The changes in NMJ innervation in superficial fibers of the TA muscle of Thy1-YFP mice were assessed at 1, 3, 7 and 10 days post-nerve transection (Fig. 1e). NMJs prior to denervation showed the characteristic “pretzel” pattern in which pre-synaptic and post-synaptic regions were fully overlapped (i). At 24 h post-nerve transection there was clear disruption of the NMJs (ii), with loss of pre-synaptic structure and axonal breakdown. By 7 days no pre-synaptic axonal structure was visible (iii). Throughout the time course the post-synaptic AChR clusters, remained relatively intact although by 10 days there was some spreading and dissolution of the AChR and multiple small areas of α-bungarotoxin staining were apparent distant from the residual AChR (iv).

Up-regulation of neural cell adhesion molecule (NCAM) expression in muscle fibers is routinely used as a clinical marker of previous denervation in pathological studies. In fully innervated TA muscle NCAM staining was detected predominantly around the nerve branches and in the muscle fiber membrane. Following transection little NCAM staining was present, but positive NCAM staining within fibers was evident only in some fibers by 7 and 10 days (Supplementary Fig. S1).

### 3.2. Mitochondrial H_2_O_2_ release from muscle fibers following full transection of the peroneal nerve

**Fig. 2:**
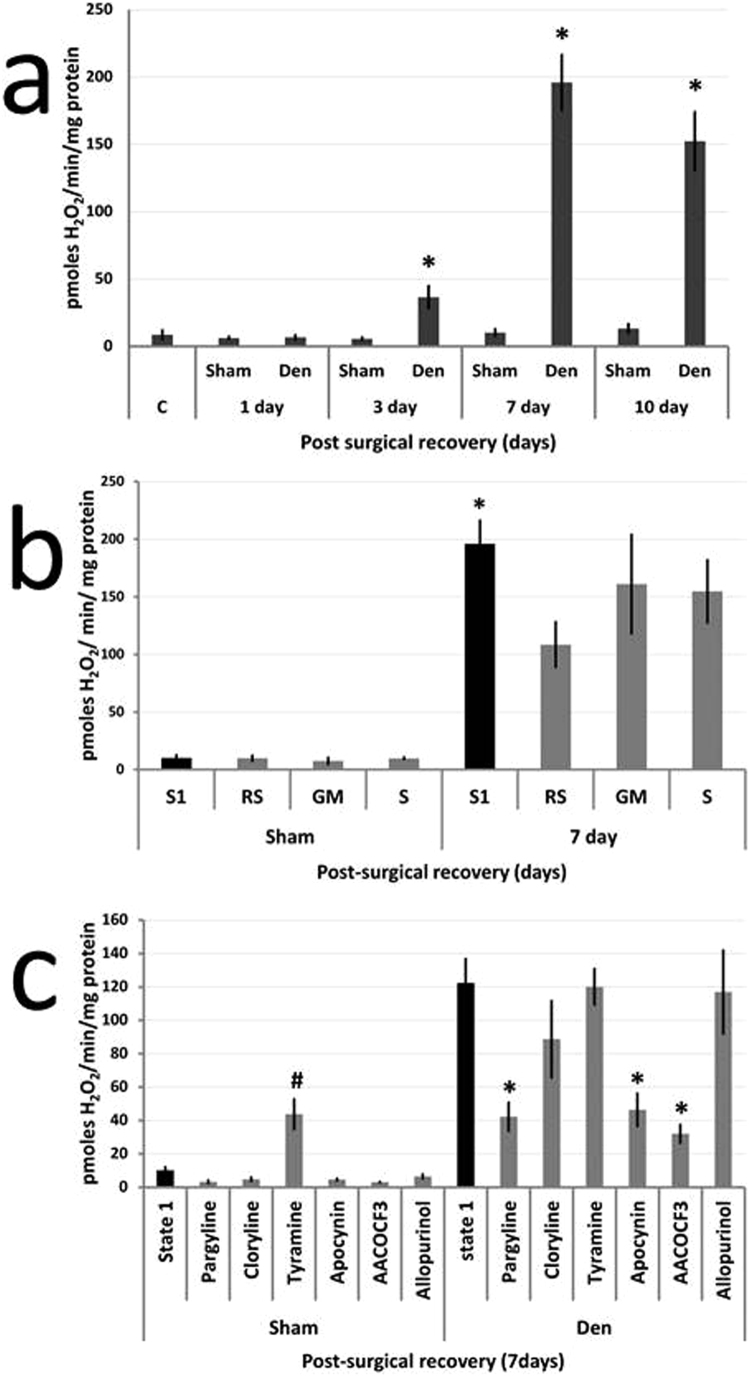
**Effect of peroneal nerve transection on peroxide generation by mitochondria from TA muscles.** The rate of mitochondrial H_2_O_2_ production by permeabilised bundles of fibers from the TA assessed using the amplex red assay. Following surgical transection of the peroneal nerve there was a gradual increase in the rate of production observed in state 1, which was significant at 3 days and increased further at 7 and 10 days post-surgery; *P < 0.05 compared with sham-operated control muscle (A). Generation from mitochondria in the sham-operated muscle was unchanged compared with that from naïve muscle (labelled c in Fig. 2a). Fiber bundles at 7 days post-denervation were incubated with ETC substrates glutamate and malate (GM) or succinate (S) or with an inhibitor, rotenone (RS) but these induced no significant changes in peroxide generation compared with denervated fibers in state 1; *P < 0.05 compared with sham-operated fibers in state 1 (B). Permeabilised fibers from the denervated muscle at 7 days post-denervation were incubated with inhibitors of alternative sources for peroxide generation: pargyline (MAO-B), chlorgyline (MAO-A), apocynin (NADPH oxidase) AACOCF_3_ (phospholipase A2), allopurinol (xanthine oxidase) and with a potential substrate for MAO (tyramine). Pargyline, apocynin and AACOCF_3_ significantly reduced the peroxide production compared with state 1, *P < 0.05 compared with denervated fibers in state 1. Tyramine significantly increased peroxide generation from permeabilised fibers from the contralateral sham-operated muscle, #P < 0.05 compared with sham-operated muscle fibers in state 1 (C).

A large increase in the rate of oxidation of amplex red was observed with bundles of permeablised TA fibers following full denervation. The peroxidase-catalysed oxidation of amplex red reflects mitochondrial production of H_2_O_2_ and potentially other peroxides. Data in Fig. 2a indicate that the peroxide release was higher than from fibers from the contralateral sham-operated TA muscle by 3 days post-surgery (p < 0.05) and remained elevated until at least 10 days (p < 0.05). Mitochondrial H_2_O_2_ production did not differ at any time point in fibers from the contralateral sham-operated TA muscle compared with control mice (Fig. 2a).

Data in Fig. 2a show mitochondrial production in state 1. Mitochondrial H_2_O_2_ generation is thought to primarily occur through dismutation of superoxide generated under specific bioenergetic conditions at sites in the ETC. Superoxide is converted to H_2_O_2_ in the mitochondrial matrix or intermembrane space (IMS) and superoxide dismutase (SOD) is added to the reaction mix to ensure complete conversion to H_2_O_2_ for detection. In order to determine the ETC site contributing to the superoxide generation presumed to be induced by denervation, fiber bundles were treated with complex I substrates (glutamate plus malate), the complex II substrate (succinate) that drives superoxide generation through both complexes I and III, and succinate plus rotenone, an inhibitor of reverse transfer of electrons to complex I [29]. In permeabilised TA fibers from sham-operated mice addition of succinate increased mitochondrial H_2_O_2_ generation and the succinate driven generation was reduced by rotenone as anticipated (Fig. 2b), but in the fibers from TA muscles at 3, 7 and 10 days post-denervation addition of these substrates did not increase amplex red oxidation above state 1 conditions, nor was there any significant effect of rotenone (Fig. 2b shows data for 7 days post-surgery, other time points are shown in Supplementary Fig. S2). These data support the hypothesis that the denervation-induced increase in mitochondrial amplex red oxidation was not due to H_2_O_2_ production deriving from superoxide produced by the ETC.

Other sources for mitochondrial peroxide production were therefore examined by addition of inhibitors of potential pathways involved. Previous data suggest that lipid peroxides can be released from mitochondria following denervation and oxidise amplex red [24]. This possibility was supported since the phospholipase A2 inhibitor, AACOCF3, reduced peroxide production compared with state 1 (Fig. 2c). Other studies indicate that skeletal muscle mitochondria contain isoforms of NADPH oxidase [30] and addition of apocynin (a NADPH oxidase inhibitor) significantly reduced the denervation-induced amplex red oxidation. Similarly skeletal muscle has been reported to contain monoamine oxidase (MAO) B [31] and pargyline (a MAO B inhibitor) also reduced the denervation-induced oxidation of amplex red. No effect was seen with chlorgyline (a MAO A inhibitor) or allopurinol (a xanthine oxidase inhibitor). These inhibitors and substrates were also added to fiber bundles from the contralateral sham-operated TA and tyramine (a MAO substrate) increased amplex red oxidation (Fig. 2c).

### 3.3. Time course of changes following partial transection of the peroneal nerve

**Fig. 3:**
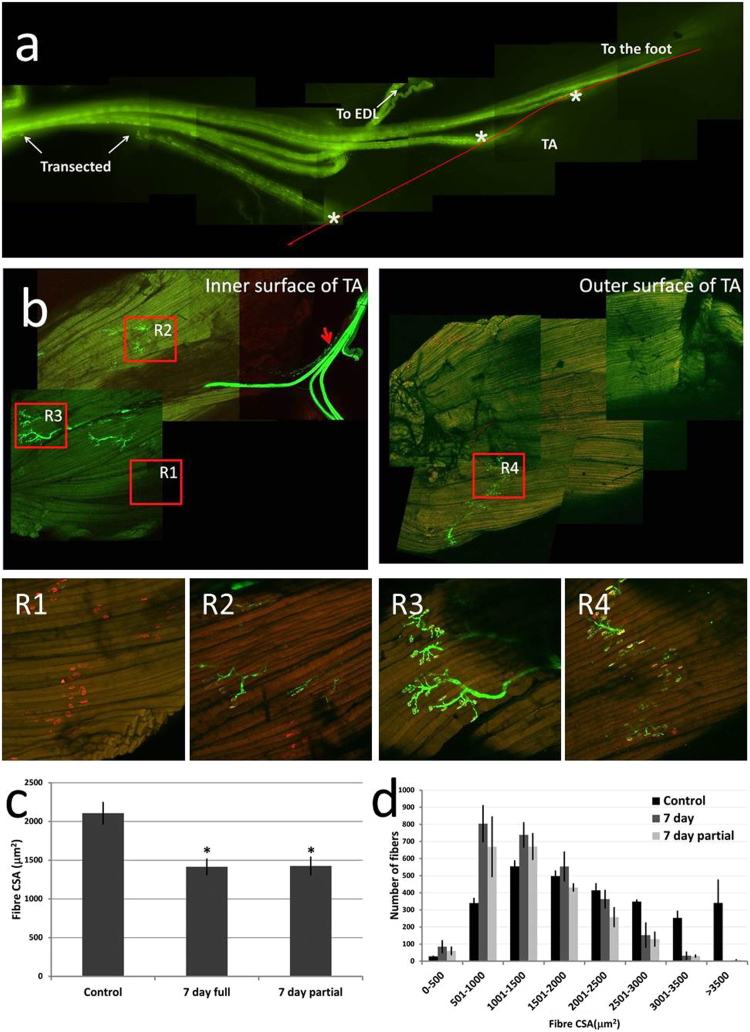
**Effect of transection of one branch of the peroneal nerve on structure of the TA muscle.** Use of the Thy1-YFP mice allowed one of the 3 branches of the peroneal nerve (indicated by * in the figure) to be transected immediately prior to entry into the TA muscle leaving 2 branches intact. This image was taken at 7 days post-transection showing lack of any regrowth of the nerve (A). Intact muscles stained with α-bungarotoxin to visualise the AChR showed variability in NMJ structure (B). At 7 days post-surgery, four regions were identified where: all fibers had lost axonal input but retained AChR (R1), all fibers retained full innervation (R3), or fibers had a mix of innervated and some denervated fibers (R2, R4). The effect of the partial denervation on muscle fiber CSA at 7 days post-surgery was similar to that observed following full denervation; *P < 0.05 compared with control non-denervated muscles. (C). The change in distribution of fiber CSA was similar to that following full denervation (D).

The observation that, by 7 days post-denervation, the AChR remained visible with no evidence of pre-synaptic input allowed identification of the innervation status of individual muscle fibers in different regions of the TA muscle following transection of one of the three branches of the peroneal nerve entering the TA (Fig. 3a). There was considerable variability in axonal and NMJ integrity between regions of the muscle surface as shown in Fig. 3b. Thus in region R3 there was normal innervation with only fully occupied NMJs visible throughout, whereas in R1 no pre-synaptic structure was visible with only staining of the AChR apparent. In contrast, in fibers from other regions (R2, R4) some NMJs showed pre-synaptic withdrawal while others still were intact. The partial denervation protocol caused a similar decrease in average fiber CSA to that seen following full denervation of the TA (Fig. 3c) that was primarily due to a reduction in the numbers of large fibers.

### 3.4. Mitochondrial peroxide release from muscle fibers following partial denervation of the TA muscle

**Fig. 4:**
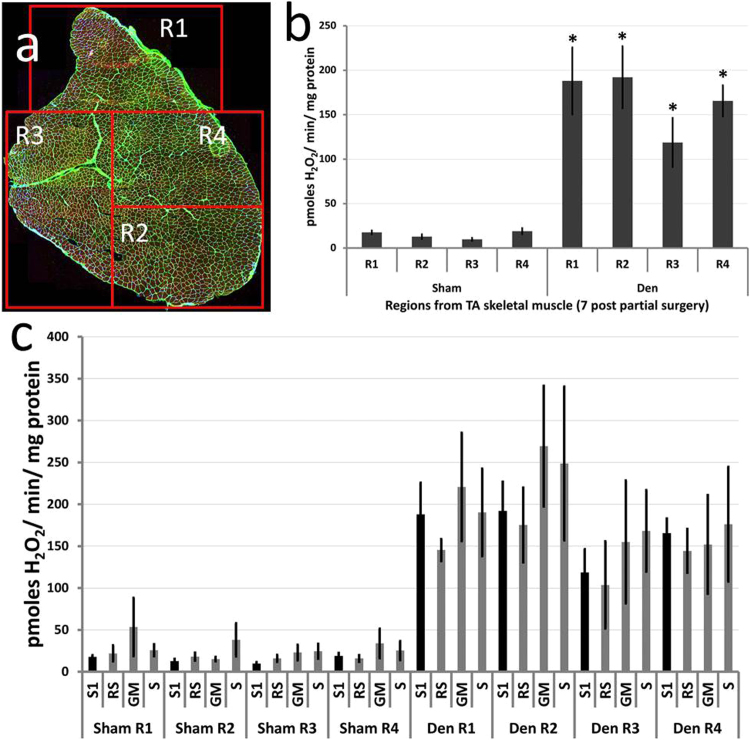
**Effect of partial denervation of the TA muscle on peroxide generation by mitochondria from different regions of the muscle.** The regions identified in Fig. 3 in longitudinal section were identified on transverse sections of the TA muscle and small bundles of fibers were obtained from each region (A). These fibers were permeabilised and state 1 mitochondrial peroxide generation examined at 7 (B) days post-surgery in comparison with sham-operated control muscles; *P < 0.05 compared with fibers from the same region of sham-operated muscles. Fiber bundles from the 4 regions of the TA were also incubated with ETC substrates (GM or S) and the inhibitor of the ETC (RS) and mitochondrial peroxide generation examined. There were no significant differences in peroxide release between any of the denervated groups following treatments (C).

Fig. 3b shows the variation in innervation status of NMJs across 4 regions of the inner and outer surfaces of the TA muscle and Fig. 4a shows the location of these same regions in a transverse section of the TA. The muscle was divided to obtain small bundles of fibers from regions with differing innervation status. Bundles of fibers from regions that showed full denervation (R1), full innervation (R3) and a mix of innervated and denervated fibers (R2 and R4) were permeabilised and mitochondrial peroxide generation examined. At 7 days (Fig. 4b) post-surgery the oxidation of amplex red by mitochondria was significantly increased in all regions over that from fibers in the same regions of the control TA muscle (p < 0.05) with no significant differences between the different regions. Addition of the ETC substrates glutamate and malate or succinate and the inhibitor rotenone had no significant effect on peroxide release in fibers from all regions (Fig. 4c) indicating that the increased ROS detected from mitochondria in all fibers was unlikely to be derived from the ETC.

### 3.5. Mitochondrial peroxide release in muscle fibers from old mice following full transection of the peroneal nerve

**Fig. 5:**
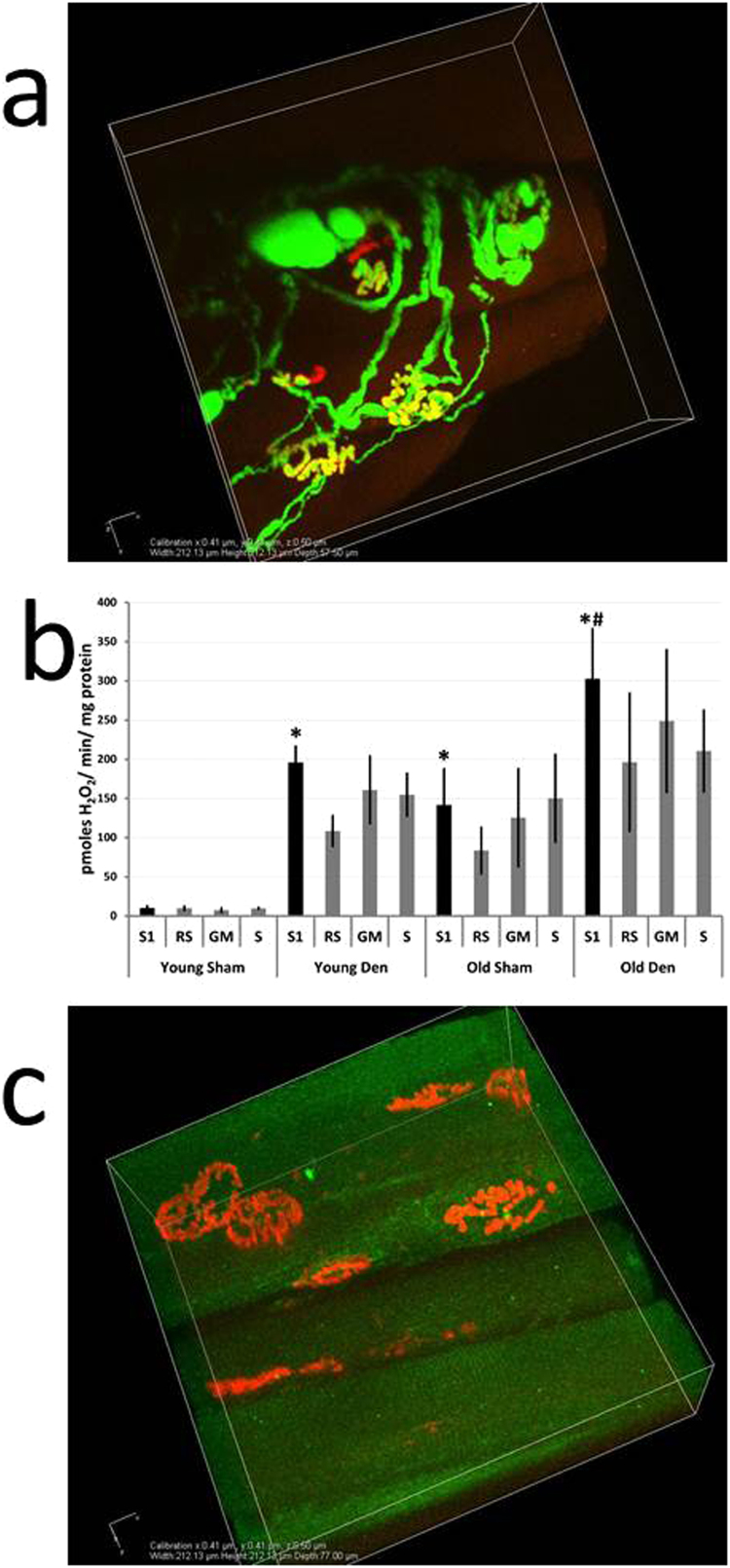
**Effect of peroneal nerve transection on peroxide generation by mitochondria from TA muscles of adult and old mice.** The structure of the NMJ in old mice appeared fragmented with examples of axonal blebs and bulging and some incomplete synaptic overlap (A). The rate of mitochondrial H_2_O_2_ production from permeabilised fiber bundles from the TA muscle at 7 days post-transection of the peroneal nerve in adult and old mice. Fiber bundles from the groups of adult and old mice were also incubated with ETC substrates (GM or S) or the ETC inhibitor (RS) and mitochondrial peroxide generation examined in comparison with sham-operated mice at the same ages (B); *P < 0.05 compared with fibers from sham-operated muscles from young mice in state 1; #P < 0.05 compared with fibers from sham-operated muscles from old mice in state 1. Following full denervation of the TA muscle there was no evidence of axonal input in the TA from old mice (C).

The NMJs in muscle fibers from sham-operated mice of 24 months of age (Fig. 5a) showed the age-related changes in NMJ structure previously reported [7], [8], [9], [10]. Mitochondrial peroxide production was elevated from bundles of fibers from the TA muscles of old mice without any experimental denervation (i.e. the sham-operated muscles) in comparison with non-denervated muscles from adult mice (Fig. 5b). In a similar manner to the denervated muscle fibers from young mice, addition of the ETC substrates glutamate and malate, or succinate and the inhibitor rotenone indicated that the increase seen in TA fibers from old mice was unlikely to be related to increased generation from ETC complexes. Full experimental denervation of the TA muscle from old mice, confirmed by lack of any axonal input (Fig. 5c), led to a further increase in mitochondrial peroxide production compared with fibers from sham-operated old mice, but the overall levels reached were not significantly different to those from denervated fibers of adult mice.

## 4. Discussion

Here we report that the presence of a small number of recently denervated fibers in a skeletal muscle leads to increased mitochondrial generation of peroxides by the denervated fibers and by neighboring innervated fibers and that this increase does not appear to require increased ROS generation by the ETC. These data provide a novel explanation for the increased mitochondrial oxidative stress and damage seen in both innervated and denervated fibers in aging skeletal muscles.

Following full denervation of the TA muscle, analyses of mitochondrial peroxide generation indicated a significant increase from fiber bundles by 3 days post-denervation (Figs. 2 and S2). Comparisons with the data on structural changes indicate that this increased release occurred in the presence of intact post-synaptic AChR with no evidence for re-innervation of the muscle (Fig. 1e) and preceded significant fiber atrophy (Fig. 1c). Our original experimental plan envisaged developing a model to allow partial denervation of a specific region of the TA muscle and that area would be defined by demonstration of NCAM expression as a marker of denervation [21], [32], but the data obtained following full peroneal nerve transection indicated the unreliability of NCAM expression as a marker of previous denervation (Fig. S1). Use of the Thy1-YFP mouse facilitated identification and transection of a single branch of the peroneal nerve that innervates part of the TA muscle with little collateral tissue damage, but also allowed clear assessment of the integrity of the peripheral motor axons and NMJ in detail. The partially denervated TA contained areas where all fibers were denervated, all fibers remained innervated, or contained a mixture of denervated and innervated fibers. The relatively widespread distribution of fibers in the TA muscle innervated by only a minor branch of the peroneal nerve is potentially advantageous to the animal in minimising the deleterious functional effects of any nerve damage. Transection of the single branch of the peroneal nerve induced a decline in the average muscle fiber area in the TA muscle that was similar to that seen following transection of the full peroneal nerve and in both models the predominant effect was a decline in the number of larger fibers (Fig. 1, Fig. 3).

Bundles of fibers were obtained from all 4 regions, permeabilised and mitochondrial H_2_O_2_ production examined. Mitochondrial peroxide generation was increased in all regions in comparison with similar regions from the sham-operated control TA muscle at 7 days post-partial denervation and there was no significant difference in the release between the different regions at either time point (Fig. 4). Thus partial denervation of the TA muscle caused an increase in mitochondrial peroxide generation and fiber atrophy in both denervated and innervated fibers. Although all fibers in region R3 shown in Fig. 4 appeared to retain innervation, a potential explanation for the data obtained could be that a small number of denervated fibers were included in each bundle, but the lack of detection of any superficial fibers in region R3 showing denervation and the relatively small number of fibers included in each bundle (15–20 fibers) argues against this explanation. An alternative explanation is that recently denervated muscle fibers or transected nerve generate signals that stimulate all muscle fibers to increase mitochondrial peroxide generation. There is some evidence from non-mammalian systems that support this possibility since Gauron et al. [33] have reported that nerves control redox levels in mature tissues in a zebrafish fin amputation model. Further they have identified Schwann cells and Hedgehog signalling as effectors of the changes in H_2_O_2_ production that occurs following section of sensory neurons. The potential role of Schwann cells and Hedgehog signalling in the increased mitochondrial H_2_O_2_ production seen from innervated or denervated fibers in the current denervation model is unknown.

The time course of the increase in mitochondrial H_2_O_2_ production following either full or partial denervation of the peroneal nerve is compatible with peroxides playing a role in the subsequent muscle fiber atrophy through stimulation of apoptosis or autophagy as originally proposed [19]. An alternative possibility is that the increased mitochondrial peroxide generation may reflect an end-organ response to stimulate re-innervation through promotion of axonal sprouting and nerve regrowth since studies in zebrafish have indicated that H_2_O_2_ promoted axonal regeneration in skin [34] and a study in mammalian neurons indicated that physiological levels of H_2_O_2_ stimulated cell proliferation [35].

We report here that the denervation-induced increase in muscle mitochondrial peroxide production does not appear to require superoxide generation from ETC since neither addition of substrates linked to ETC complexes I or II nor use of an inhibitor (rotenone) caused any significant increase or decrease in peroxide production. In contrast inhibitors of MAO B, NADPH oxidases and phospholipase A2 significantly reduced the post-denervation increase in muscle mitochondrial peroxide production. In studies following the original description of increased muscle mitochondrial peroxide release following denervation [19], the same group of researchers reported that lipid peroxides were also released from muscle mitochondria following denervation and react with amplex red and thus the amount of H_2_O_2_ released may be overestimated [24]. They further reported that release of these peroxides was mediated by activation of phospholipase A2 and that they may play a role in the subsequent denervation-induced muscle fiber atrophy. As part of the current work we also examined the effect of catalase (50 U/ml) or sodium pyruvate (1 mM) as scavengers of H_2_O_2_ on oxidation of amplex red by permeabilised fibers obtained from muscles at 7 days post-denervation. Both of the scavengers reduced the amplex red oxidation by ~ 43% from fibers in state 1 respiration (data not shown in detail) which is broadly comparable to the contribution of H_2_O_2_ to amplex red oxidation in denervated muscle fibers reported by Bhattacharya et al. [24]. In the current paper we have therefore described the compounds that are released by mitochondria and react with amplex red as peroxides to acknowledge the potential contribution of both H_2_O_2_ and lipid peroxides. Both of these compounds can diffuse within the cell and affect redox-responsive pathways and induce oxidative damage. Our data do not support an effect of denervation to increase muscle mitochondrial ROS generation through the ETC, but are compatible with involvement of other peroxide generating systems found within mitochondria. Skeletal muscle mitochondria have previously been shown to express MAO B [31], NADPH oxidase 4 [30] and phospholipase A2 [24].

The use of isolated permeablised muscle fibers provides some reassurance that the mitochondria derived from muscle in comparison with previous studies where mitochondria were isolated from whole muscles that would contain non-myogenic cells [19]. In addition the fiber bundles were washed multiple times in relax solution with and without saponin prior to study removing blood and inflammatory cells from the preparation.

Mitochondrial peroxide release from muscle fibers from the TA muscles of old mice without experimental denervation was increased in state 1 compared with adult mice as previously reported [17]. The mechanisms underlying this increase in basal mitochondrial peroxide release in skeletal muscle from old mice has not been determined although an age-related increase in angiotensin II has been implicated in the muscle loss acting potentially through increased NADPH oxidase or mitochondrial ROS generation [36]. In the current study we showed that following denervation the level of peroxide production from fibers from old mice increased further to levels that were not significantly different to those seen from denervated fibers from adult mice. Addition of ETC substrates indicated that similar non-ETC mechanisms appeared to contribute to the peroxide generation in old mice pre-and post-denervation as in the adult mice post-denervation. During aging, muscle fiber loss is associated with a loss of motor units [5], [37]. Aging is also associated with numerous pre- and post-synaptic structural abnormalities [38], [39] and fragmentation of postsynaptic motor endplates [6], [9]. Thus even within a single motor unit in aged mice some NMJ retain full innervation whilst others show disruption and lack of pre-synaptic input [6]. The data presented here indicate that during aging this loss of innervation of individual fibers will play a major role in increasing mitochondrial peroxide production in neighboring fibers in the muscle and contribute to the substantial increase in mitochondrial peroxide generation that has been previously reported in muscle from aged rodents [17].

In conclusion, here we report that denervation of muscle leads to a large increase in release of H_2_O_2_ and lipid peroxides from muscle mitochondria over a time course that precedes loss of muscle mass. Furthermore we show that the presence of a small number of recently denervated fibers in a skeletal muscle leads to increased mitochondrial peroxide generation by neighboring innervated fibers. The mechanisms by which this increased release occurs do not appear to involve generation of ROS from the ETC but may require monoamine oxidase, NADPH oxidase and phospholipase A2 activities. These data provide a novel explanation for the increased mitochondrial oxidative stress and damage seen in aging skeletal muscles.

## Author contributions

N.P, C.S. and A.V planned and conducted the experiments; N.P. and A.V. planned and undertook the surgery and analysed the data; N.P., M.J.J. and A.McA. designed the study and wrote the paper.

## Competing financial interests

The authors declare no competing financial interests.
