# Supplementary material for: Denervated muscle fibers induce mitochondrial peroxide generation in neighboring innervated fibers: Role in muscle aging
Source: Free Radic Biol Med. 2017 Nov;112:84–92. doi: 10.1016/j.freeradbiomed.2017.07.017 (PMC5636617; doi:10.1016/j.freeradbiomed.2017.07.017)
Supplement: Supplementary file 1 — Supplementary material [file mmc1.docx]

# Denervated muscle fibers induce mitochondrial peroxide generation

# in neighbouring innervated fibers: Role in muscle aging

Natalie Pollock, Caroline A Staunton, Aphrodite Vasilaki, Anne McArdle, Malcolm J Jackson*

**Supplementary data**

**Figure S1.** Expression of NCAM in transverse sections of the denervated TA muscle. Minimal staining was observed located in and around the nerve in control tissue. At 1 and 3 days following full transection of the peroneal nerve staining was very weak but at 7 and 10 days NCAM was expressed within a small proportion of muscle fibers.

**Figure S2.** The production of peroxides from mitochondria in permeabilised muscle fibers at 1, 3, 7 and 10 days following transection of the peroneal nerve. Data are shown for fibers in state 1 or incubated with ETC substrates glutamate and malate (GM), succinate (S) or with succinate plus rotenone (RS); *P<0.05 compared with fibers from control sham-operated muscles in state 1. No significant effects of the ETC substrates or inhibitor were seen on denervated fibers compared with peroxide production in state 1 at the same time point.

**Figure S1**

**
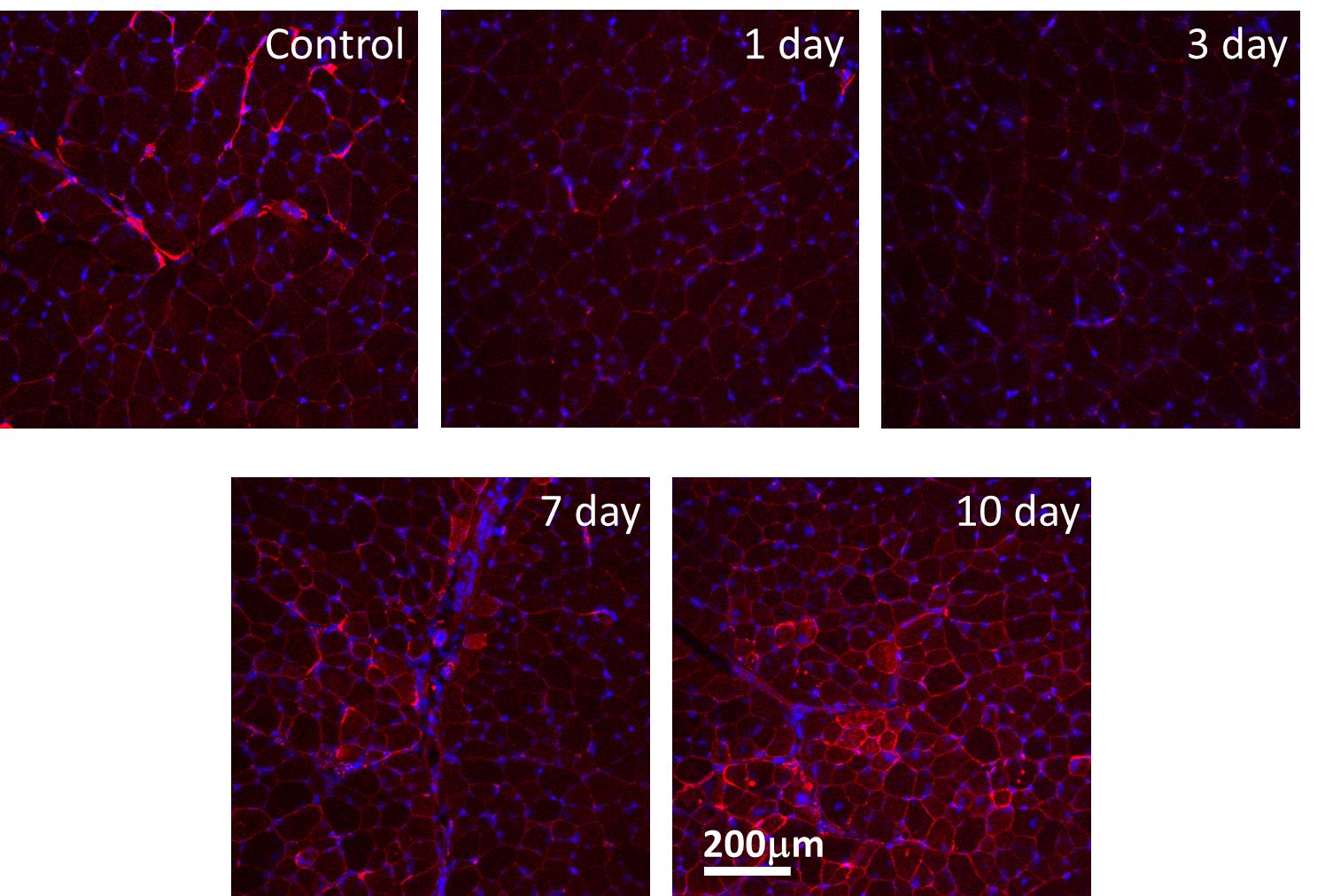
**

**Figure S2**

**
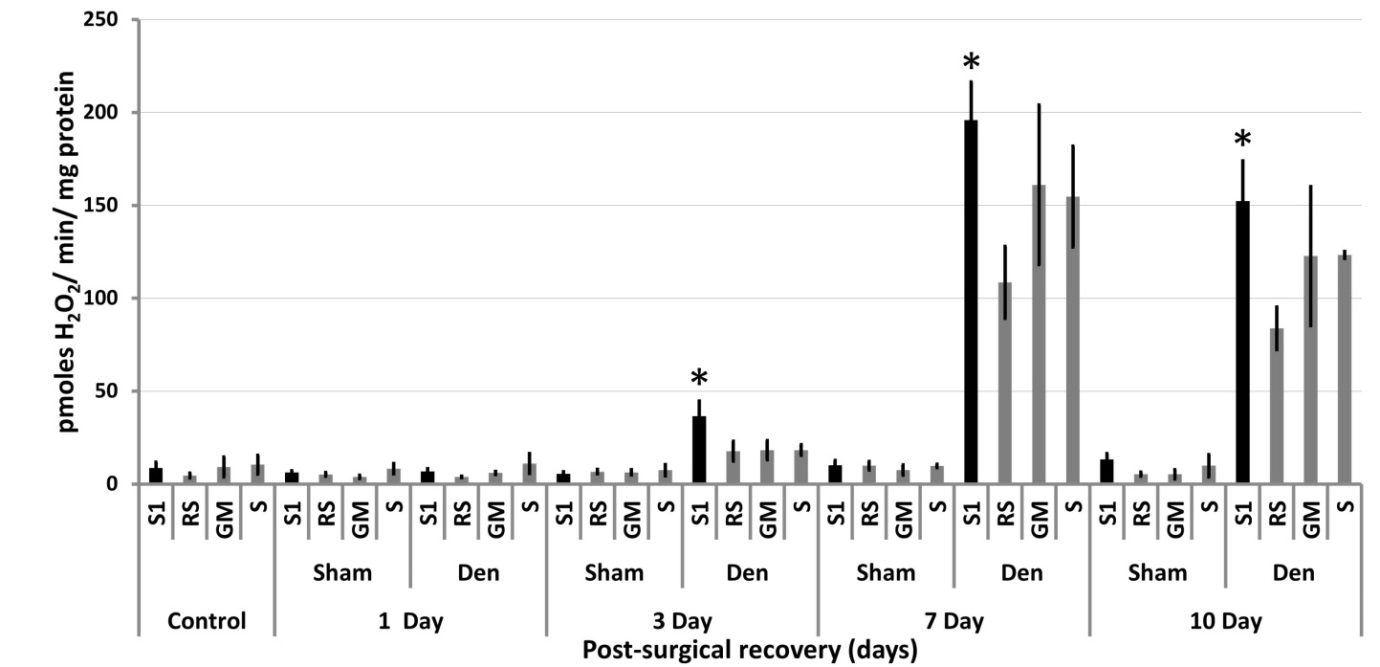
**
